# Correlation Between Angular Position and Pathological Changes in Impacted Lower Third Molars: A Systematic Review and Meta-Analysis

**DOI:** 10.3390/dj13030129

**Published:** 2025-03-14

**Authors:** Shaniko Kaleci, Pierantonio Bellini, Giacomo Setti, Giulia Melloni, Matilde Ruozzi, Ugo Consolo

**Affiliations:** Department of Surgery, Medicine, Dentistry and Morphological Sciences with Transplant Surgery, Oncology and Regenerative Medicine Relevance (CHIMOMO), University of Modena and Reggio Emilia, 41125 Modena, Italy

**Keywords:** impacted third molars, pathological changes, mesioangular position, meta-analysis, alveolar bone loss

## Abstract

**Background:** The extraction of impacted third molars presents anatomical challenges and surgical risks, prompting debate over prophylactic removal, particularly for impacted lower molars. Studies highlight associated pathologies and complications that influence treatment decisions. **Objective:** This study aims to systematically review and analyze the correlation between the angular positions of impacted lower third molars and their association with pathological changes, including periodontal defects, alveolar bone loss, and cystic degeneration. **Methods:** This systematic review was conducted according to PRISMA guidelines, including studies from 2000 to 2024. Studies reporting the angular position and associated pathologies of impacted lower third molars were included. The Newcastle-Ottawa Scale (NOS) was used to assess the risk of bias. A meta-analysis of the proportion of pathological changes related to specific angular positions was performed. **Results:** Of the 2943 studies initially identified, six studies (including 2222 patients and 3276 impacted lower third molars) met the inclusion criteria and were included in the review, while four studies were included in the proportional meta-analysis. The most common angular positions observed were mesioangular (34.0–66.1%), followed by vertical (12.8–48.3%), horizontal (8.1–23.4%), and distoangular (3.2–14.0%). Pathological changes were reported in 8.1–75.0% of cases, with horizontal impactions demonstrating the highest correlation (41.1%, 95% CI: 20.9–63.0). **Conclusions:** The angular position of impactions of the lower third molars significantly influences the risk of pathological changes. Horizontal impactions exhibit the highest associated with pathological changes, whereas distoangular impactions show the lowest prevalence of complications. Standardized diagnostic and reporting practices are needed to improve clinical decision-making. Further research should focus on long-term outcomes and the impact of clinical management strategies.

## 1. Introduction

The surgical removal of impacted third molars is among the most commonly conducted procedures in oral surgery practices. Private clinics often prefer to refer their patients to university clinics or hospitals due to the intrinsic operational difficulty of the intervention and the professional risk involved. Surgery involving the third molars can present intraoperative challenges and complex postoperative patient management, potentially requiring numerous appointments for dressings. Complications related to various surgical and anatomical factors can occur. Recent studies emphasize the growing need for better preoperative planning and patient selection to mitigate these risks [1,2,3,4,5,6,7].

The removal of lower third molars, in particular, is a complex surgery due to the anatomical position of the molars, proximity to vital anatomical structures, and limited operative space. The necessity of prophylactic extraction of lower third molars remains a topic of ongoing discussion [8,9], with some advocating for preventive removal to avoid future dental issues, while others argue for a more conservative approach, favoring retention of asymptomatic molars [10,11]. Despite these differing views, a significant reduction in the number of prophylactic extractions has been observed following updates to clinical guidelines in several countries.

In recent years, the number of impacted third molars removals in the UK has significantly decreased after the update of the NICE guidelines in 1997, which recommended preserving asymptomatic third molars [12,13,14]. Up until the mid-1990s, the surgical removal of impacted third molars was one of the most frequently performed procedures on young adults in the UK. This surgical procedure requires substantial human and economic resources. In 1998, American guidelines concluded that there was insufficient evidence to support the routine prophylactic extraction of asymptomatic third molars [15,16,17,18].

This shift in perspective has sparked ongoing debates, with various studies offering differing views on the benefits and risks of the procedure. Some experts argue that extracting impacted third molars could prevent potential complications like pain, infections, cysts, and tumors that could arise from impacted teeth, while others suggest that the risks associated with prophylactic surgery often outweigh the benefits. Additionally, impacted third molars frequently contribute to caries and pathological root resorptions in the second molar [19].

Furthermore, there is ongoing debate about the misalignment of anterior teeth caused by the eruption or incomplete eruption of lower third molars. Some studies have also documented the relationship between impacted molars and the development of cysts in the surrounding tissues, underscoring the importance of early clinical intervention and careful monitoring.

The background to this debate is multifaceted. Historically, the removal of impacted lower third molars was considered a routine preventive measure to avoid pain, infections, cysts, and tumors. However, recent studies and updated guidelines have questioned the necessity of prophylactic extractions, citing risks such as nerve injury, bleeding, dry socket, and post-operative complications. Conversely, untreated impacted lower third molars have been associated with a range of pathological changes, including periodontal defects, root resorption, and alveolar bone loss.

This study aims to systematically review and analyze the relationship between the angular position of impacted lower third molars and their associated pathological changes. By synthesizing available evidence, we aim to provide insights into the implications of these conditions for clinical management and treatment decisions.

## 2. Materials and Methods

The study protocol was registered in the International Prospective Register of Systematic Reviews (PROSPERO) under registration number: CRD42024536337, available at http://www.crd.york.ac.uk/PROSPERO, accessed on 15 April 2024. This systematic review and meta-analysis were conducted in accordance with the PRISMA 2020 guidelines, and the methods and results were reported in alignment with the PRISMA checklist.

The meta-analysis focused exclusively on English-language databases, with a comprehensive search conducted independently by two investigators (G.M., S.K.) in the PubMed, Web of Science, Embase and Cochrane Library databases. The search covered the literature published from the inception of each database through 30 January 2024. The review question was formulated based on the PICO (Population or Problem, Intervention or Exposure, Comparison, Outcome) framework.

We employed the following search string and consulted relevant narrative texts, including those by Chiapasco et al. (2013) [20] and the Illustrated Manual of Oral Surgery:

### 2.1. Search Strategy

The review question was framed using the PICO model:

Population: Adults aged 18 years and older.

Intervention: Extraction of impacted lower third molars.

Comparison: Retained asymptomatic lower third molars.

Outcome: Pathological changes (e.g., periodontal defects, alveolar bone loss).

(((extraction impacted lower third molar) OR (periodontal defect distal second molar)) OR (alveolar bone defect) NOT case report NOT (regeneration) NOT furcation NOT (fibula) NOT implant NOT PRF NOT PRP NOT treatment NOT DRUGS Filters: Humans, English, Adult: 18+ years 2.2)

Search Strategies: A review of the scientific literature was conducted.

### 2.2. Study Selection

The research was conducted using the Medline and PUBMED databases, and narrative texts (Chiapasco et al., 2013 [20], Illustrated Manual of Oral Surgery) were consulted. The following keywords were used:

“extraction impacted lower third molar”;

“periodontal defect distal second molar”;

“alveolar bone defect”.

The keywords and Boolean operators used to construct the search strings are as follows:

(((extraction impacted lower third molar) OR (periodontal defect distal second molar)) OR (alveolar bone defect)) NOT (case report) NOT (regeneration) NOT (furcation) NOT (fibula) NOT (implant) NOT (PRF) NOT (PRP) NOT (treatment) NOT (DRUGS) Filters: Humans, English, Adult: 18+ years

A total of 2943 articles were retrieved. Two authors independently screened titles and abstracts, resolving disagreements through consensus discussions. During the study selection process, the article by Sharma et al. (2023) [21] was not initially retrieved in our systematic search because the selected keywords (‘extraction impacted lower third molar’, ‘periodontal defect distal second molar’, ‘alveolar bone defect’) did not appear in the article title. However, this study was later identified through citation in another included article and was deemed relevant to our analysis. Therefore, it was included in the final selection to ensure comprehensive coverage of the topic.

### 2.3. Inclusion Criteria

Adult patients (aged >18 years).

Population: Human sex.

Intervention: extraction of impacted lower third molars.

Study characteristics: The literature search for this review and meta-analysis was restricted to studies published between 2000 and 2024. This time frame was chosen to ensure the inclusion of the most recent and clinically relevant data, reflecting advancements in surgical techniques, diagnostic methods, and our understanding of the pathological processes associated with impacted lower third molars. Older studies were excluded to minimize heterogeneity arising from outdated methodologies and clinical practices. While acknowledging the potential value of earlier research, the selected period allowed us to focus on evidence generated within a framework of modern clinical standards and diagnostic tools.

### 2.4. Exclusion Criteria

Articles not written in English were excluded.

All case reports were excluded due to insufficient scientific evidence, as were studies related to periodontal regenerative techniques using autologous bone or synthetic bone substitutes, both on natural teeth and on implants affected by peri-implantitis.

Out of the initial 2943 articles, 92 fulfilled the preliminary inclusion criteria as determined by both authors. These articles were retrieved and thoroughly reviewed by the same two researchers, who collaborated to resolve any disagreements regarding their inclusion.

After reading the titles, off-topic studies related to periodontology, cranial anomalies, or three-dimensional studies of alveolar bone were excluded. Thus, 12 studies were obtained; after the entire abstract was read, 5 additional studies were excluded for the following reasons: multiple presentations, orthodontic studies, and radiological studies on CBCT.

Of the 7 selected studies, 2 concerned the surgical technique of coronectomy [22,23]. Therefore, a meta-analysis was performed on 5 studies [24,25,26,27,28]. During the reading of the scientific articles, one study was removed due to a lack of data on the position of the third molar and 1 study was added to the meta-analysis because it was mentioned by Göksel et al., 2011 [27] in a table and the reported data were particularly interesting for our evaluation.

An additional manual review of the references cited in the selected articles was conducted. However, this process, along with a supplementary PubMed search, did not identify any additional studies that met the inclusion criteria. Ultimately, two reviewers independently extracted data, including:

Study characteristics (design, sample size, location).

Patient demographics (age, sex).

Key outcomes (proportion of pathological changes, complications).

Discrepancies in data extraction were resolved through discussion and consultation with a third reviewer.

### 2.5. Risk of Bias Assessment

The quality of the included studies was evaluated using the Newcastle-Ottawa Scale (NOS). The NOS criteria assess selection, comparability, and outcome domains, allowing for a comprehensive appraisal of methodological quality. Disagreements during this process were resolved by consulting a third reviewer.

### 2.6. Statistical Analysis

A proportional meta-analysis was performed using MedCalc 14.8.1 software (MedCalc Software bvba, Ostend, Belgium; http://www.medcalc.org; 2014), employing the Freedman–Tukey test to calculate weighted overall proportions with 95% confidence intervals (CIs). Both fixed-effects and random-effects models were employed to estimate the overall effects. The fixed-effects model assumes that all studies have a similar effect, with the summary effect providing an estimate based on these similar effects. On the other hand, the random-effects model assumes variability in effects across studies, with the summary analysis representing a weighted average across the studies.

A forest plot was created as a graphical representation, displaying the effect size and confidence intervals for each study. It also included the weighted effect size for pathological changes at each angular position of the third molar, along with the 95% CI. The size of the marker (square) indicates the weight of each study, with studies with smaller sample sizes carrying less weight.

Forest plots visually represented the effect sizes and confidence intervals, with the width of the diamond indicating precision. Heterogeneity was evaluated using Cochran’s Q statistic and the I^2^ index, with interpretations ranging from homogeneous (I^2^ = 0–25%) to extreme heterogeneity (I^2^ = 75–100%). Forest plots were generated to visualize the proportional meta-analysis results, including confidence intervals (CIs) and study weights.

## 3. Results

**Figure 1:**
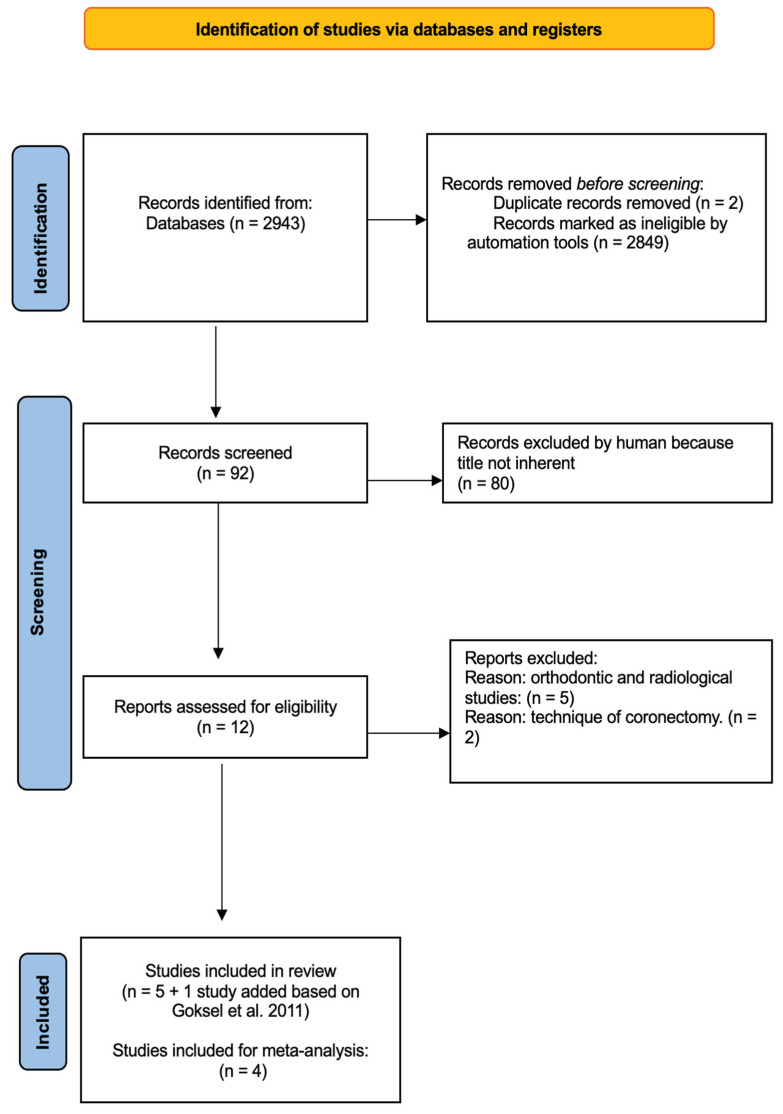
Workflow diagram depicting the systematic selection of studies for inclusion in the meta-analysis [26].

A total of 2943 studies were initially identified through the database search by two independent researchers following predefined search protocols and data collection procedures. No additional studies were retrieved from the gray literature search. After removing two duplicate studies, 2849 irrelevant studies were excluded based on titles, letters, and reviews, and 92 studies proceeded to the initial screening. After reviewing the abstracts and full texts, 80 studies were excluded because they did not meet the inclusion criteria. Ultimately, 12 studies were considered suitable for inclusion in the review and meta-analysis; of these, six studies were included into the review, and four studies were included in the proportional meta-analysis. The flow diagram illustrating the literature search process is shown in Figure 1.

Table 1 presents the detailed characteristics of the studies included in the analysis.

Six studies involving 2222 patients and four studies involving 610 patients with pathological alterations and angular tooth positions were assessed for the proportional meta-analysis. Most of the patients were female (1246; 56.1%).

Two studies did not provide specific data on the number of patients with pathological changes. Among the studies that did report pathological alterations, a total of 708 affected teeth were identified, with 124 teeth (17.7%) showing pathological changes (Table 2).

**Figure 2:**
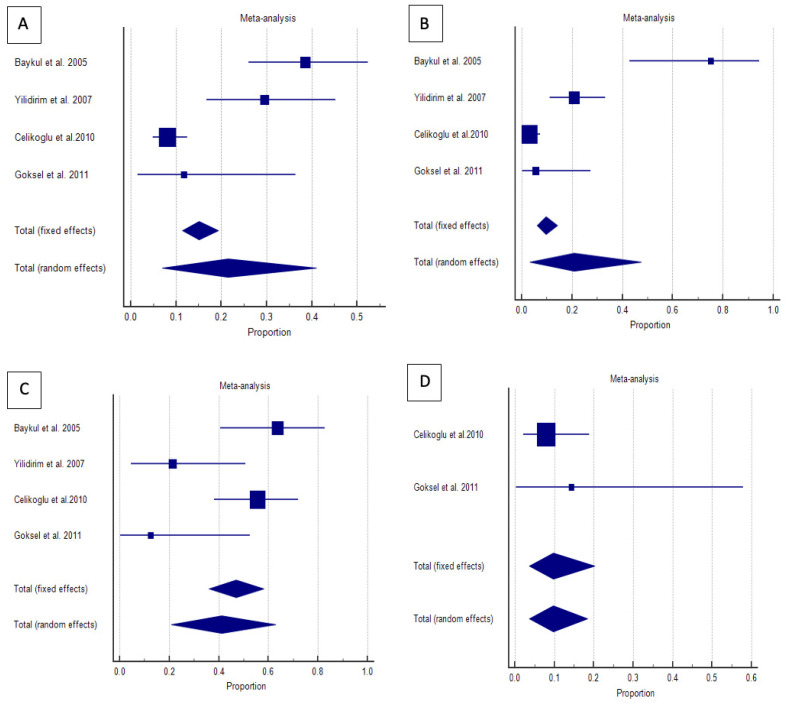
Proportional meta-analysis of included studies with pathological changes in the angular position of impacted lower third molars [(**A**) mesioangular, (**B**) horizontal, (**C**) vertical, and (**D**) distoangular] [23,24,25,26]. Aggregate percentage of patients with different frequent causes of symptoms. Markers represent grouped effects. The position of the diamond represents the estimated effect size, and the width of the diamond reflects the precision of the estimate. Output generated by MedCalc 14.8.1 software.

The correlation between pathological changes and angular position was analyzed, and proportions for each angular position of impacted lower third molars were calculated from individual studies. The results of the proportional meta-analysis, including the combined proportion (95% CI), are summarized in Table 3, with estimates of the overall proportion shown in the Forest Plot (Figure 2).

The overall proportion of impacted lower third molars in the mesioangular position was 21.4% (7.0–40.9), demonstrating significant and extreme heterogeneity (Q = 33.4, I^2^ = 91.0%, *p* < 0.001). For horizontally positioned impacted lower third molars, the overall proportion was 41.1% (20.8–63.0), indicating significant and extensive heterogeneity (Q = 11.4, df = 3, I^2^ = 73.8%, *p* = 0.009). The overall proportion of vertically positioned impacted lower third molars was 20.7% (3.4–47.4), also exhibiting significant and extreme heterogeneity (Q = 42.2, df = 3, I^2^ = 92.9%, *p* < 0.001). Conversely, the distoangular position had an overall proportion of 9.7% (3.6–18.4) and had no significant homogeneity (Q = 0.5, df = 1, I^2^= 0.0%, *p* = 0.447).

Figure 2 visually demonstrates that horizontal impactions are most strongly associated with pathological changes, whereas distoangular impactions exhibit the lowest risk. The grouped effect markers and diamond positions confirm these patterns, with the width of the diamonds reflecting the precision of the estimates.

These findings emphasize the clinical significance of angular positioning in evaluating the risks and frequency of symptoms associated with impacted lower third molars. Horizontal and mesioangular impactions may require more proactive management strategies, whereas vertical and distoangular impactions can often be managed conservatively, depending on the clinical context.

The Newcastle-Ottawa Scale (NOS) was applied to evaluate the risk of bias across the six included studies. The studies by Celikoglu et al. (2010) [26], Ryalat et al. (2018) [28], and Sharma et al. (2023) [21] received scores of 7 or higher, indicating robust study design and minimal concerns about potential bias. These studies ensured appropriate selection, comparability, and assessment criteria.

The studies by Baykul et al. (2005) [24], Yildirim et al. (2008) [25], and Göksel et al. (2011) [27] scored between 5 and 6 points, reflecting some limitations such as incomplete adjustment for confounders or limited reporting on the reliability of outcome assessment.

None of the included studies demonstrated a high risk of bias, as all met minimum standards for selection, comparability, and outcome assessment (Table 4).

## 4. Discussion

Our study identified six relevant articles in accordance with PRISMA Statement guidelines [29]. Ryalat et al., 2018 [28], investigated the optimal age for extracting impacted lower third molars, highlighting the challenge of deciding on asymptomatic extractions. Analyzing 4600 orthopantomographs from the University of Jordan, the study revealed age-related changes in third molar inclination and impaction patterns. After exclusions, the sample consisted of 1810 molars, with 1224 impacted bilaterally and 586 unilaterally. Using the William Sciller method, they demonstrated that the inclination angle of the third molar changes with age, affecting the Pell and Gregory impaction patterns. The study suggested reevaluating X-rays in younger patients to monitor changes in inclusion severity.

Baykul et al., 2005 [24], examined cystic changes in dental follicles and reported a significant association between cystic lesions and vertically impacted molars. Among the 117 selected patients, 94 were included in the study. Cystic changes were most frequent between 20 and 25 years of age, with a preference for males.

Celikoglu et al. (2010) [26] studied agenesis, impaction, positional angle, and pathological changes in 368 orthodontic patients aged >20–26 years. Agenesis of the third molars occurred in 17.3% of patients, while pathological changes, such as root resorption of the second molar and alveolar bone height reduction, occurred in 10.4% of cases. Horizontal impactions were associated with a higher frequency of pathological changes.

Göksel et al., 2011 [27], analyzed dental follicles in 50 asymptomatic patients with included or semi-included lower third molars. They concluded that radiographic evaluation alone is insufficient, as asymptomatic third molars may still cause pathological degeneration.

Yamaoka et al., 1997 [30], emphasized the risk of inflammation in completely impacted lower third molars, while Simşek-Kaya et al., 2011 [31], and Yildirim et al., 2008 [25], highlighted the potential for soft tissue pathologies and other complications, even in asymptomatic cases.

Chiapasco et al., 2013 [20], and Kugelberg et al., 1985 [32], observed a close association between pericoronitis and impacted lower third molars (95%), attributed to anatomical characteristics such as the absence of keratinized gingiva on the distal wall, which facilitates bacterial colonization and deep pocket formation. Saravana and Subhashraj, 2008 [33], underscored the necessity of clinical transformations.

Adelsperger et al. (2000) [34] noted early soft tissue pathologies around impacted third molars, emphasizing the need for detailed clinical evaluations. Kan et al. (2002) [35] identified persistent periodontal defects on the distal aspect of second molar postextraction, suggesting the need for extended monitoring and care.

Our statistical analysis revealed a greater incidence of pathological change in horizontally impacted lower third molars (41%) compared to the vertical (21.4%), mesioangular (20.7%), and distoangular (9.7%) positions. This pathological change often results in increased radiographic translucency and alveolar bone loss adjacent to the second molar. Additionally, the frequency of cyst degeneration and bone loss distal to the second molar is notably higher than previously reported, especially in patients aged 20–25 years.

Histological analysis is recommended for follicles with a radiographic width greater than 2.5 mm (accounting for magnification factor of radiographic examination) after tooth removal. Close monitoring of asymptomatic impacted lower third molars or those with reduced follicle sizes on radiographs is essential to detect complications early.

The overall quality of the included studies ranged from moderate to low risk of bias, supporting the validity of the findings but necessitating caution in interpretation due to potential confounding factors and incomplete reporting. The significant heterogeneity across studies highlights the need for high-quality research with comprehensive reporting.

The limitations of this review include the lack of long-term follow-up studies (exceeding five years) and insufficient subgroup analyses based on factors like age, sex, and comorbid conditions. Long-term studies are essential to understand chronic complications of retained lower third molars. Future research should also emphasize the use of advanced imaging modalities, such as cone-beam computed tomography (CBCT), to improve patient outcomes.

## 5. Conclusions

The findings of this systematic review and meta-analysis confirm that the angular position of impacted lower third molars significantly influences the risk of pathological changes. Horizontal impactions demonstrate the highest prevalence of alveolar bone loss and periodontal defects, while distoangular impactions show the lowest risk. These findings emphasize the importance of individualized risk assessment in the management of impacted third molars.

Several periodontal defects are associated with impacted lower third molars, including increased contact with adjacent teeth, pathological conditions affecting the alveolar bone, and inflammatory changes linked to the depth of impaction. Impacted lower third molars can significantly affect periodontal health and necessitate careful assessment and long-term monitoring

Studies have also reported a relationship between pericoronitis and impacted lower third molars, largely attributed to anatomical factors such as the absence of keratinized gingiva, which facilitates bacterial colonization and periodontal pockets formation, increasing the risk of infection. These findings highlight the importance of considering anatomical characteristics when evaluating the risks associated with impacted lower third molars.

This study highlights the prevalence of different angular positions of impacted lower third molars and their correlation with pathological changes. A horizontal position of impacted lower third molars demonstrated the highest frequency of complications, including radiographic translucency and alveolar bone loss, whereas distoangular positions showed the lowest prevalence. It is important to note that bone loss should ideally be assessed after a healing period of approximately six months post-extraction to account for bone remodeling and tissue regeneration. The higher risk of pathological changes associated with horizontally positioned impacted lower third molars warrants close monitoring and management.

Furthermore, our analysis highlights the need for standardized diagnostic criteria to improve comparability across studies and enhance clinical decision-making. Future research should focus on long-term outcomes, the impact of different management strategies, and the use of advanced imaging techniques to refine diagnostic and therapeutic approaches.

## Figures and Tables

**Table 1:** Demographic and clinical characteristics of the included studies.

Study	Pts	Lower Third Molars	Age, Mean	Male	Female
Baykul et al., 2005 [24]	94	94	21, 1	30	64
Yilidirim et al., 2007 [25]	115	120	24, 74	38	77
Celikoglu et al., 2010 [26]	351	444	22, 8	153	198
Göksel et al., 2011 [27]	50	50	21, 0	22	28
Ryalat et al., 2018 [28]	1198	1810		566	632
Sharma et al., 2023 [21]	414	758	22, 4	167	247

**Table 2:** Summary of angular positions of impacted lower third molars and the frequency of associated pathological changes, including bone loss, periodontal defects, cystic degeneration, and root resorption across studies.

Study	Lower Third Molars	Mesioangular	Horizontal	Vertical	Distoangular
Baykul et al., 2005 [24]	94	57 (60.6)	22 (23.4)	12 (12.8)	3 (3.2)
Yilidirim et al., 2007 [25]	120	44 (36.7)	14 (11.7)	58 (48.3)	4 (3.3)
Celikoglu et al., 2010 [26]	444	222 (50.0)	36 (8.1)	135 (30.4)	51 (11.5)
Göksel et al., 2011 [27]	50	17 (34.0)	8 (16.0)	18 (36.0)	7 (14.0)
Ryalat et al., 2018 [28]	1810	1196 (66.1)	273 (15.1)	340 (18.8)	
Sharma et al., 2023 [21]	758	344 (45.4)	126 (16.6)	249 (32.8)	39 (5.1)
Pathologic changes					
Baykul et al., 2005 [24]	94	22 (38.6)	14 (63.6)	9 (75.0)	
Yilidirim et al., 2007 [25]	120	13 (29.5)	3 (21.4)	12 (20.7)	
Celikoglu et al., 2010 [26]	444	18 (8.1)	20 (55.6)	4 (3.0)	4 (7.8)
Göksel et al., 2011 [27]	50	2 (11.8)	1 (12.5)	1 (5.6)	1 (14.3)

**Table 3:** Meta-analysis of the aggregated pathological changes, including bone loss, periodontal defects, cystic degeneration, and root resorption across angular positions of impacted lower third molars.

	Sample Size	n. Event	Proportion (%)	95% CI
Pathologic Changes				
Mesioangular	340	55	21.4	7.0 to 40.9
Horizontal	80	38	41.1	20.9 to 63.0
Vertical	223	26	20.7	3.4 to 47.4
Distoangular	58	5	9.7	3.6 to 18.4

**Table 4:** Risk of Bias Assessment of the included studies.

Study	Selection (4 pts)	Comparability (2 pts)	Outcome (3 pts)	Total Score	Risk of Bias
Baykul et al., 2005 [24]	3	1	2	6	Moderate
Yildirim et al., 2008 [25]	3	1	2	6	Moderate
Celikoglu et al., 2010 [26]	4	2	3	9	Low
Göksel et al., 2011 [27]	3	1	2	6	Moderate
Ryalat et al., 2018 [28]	4	2	3	9	Low
Sharma et al., 2023 [21]	3	2	3	8	Low
